# Supplementary figures and images for: Exploring Microorganisms from Plastic-Polluted Sites: Unveiling Plastic Degradation and PHA Production Potential
Source: Microorganisms. 2023 Dec 3;11(12):2914. doi: 10.3390/microorganisms11122914 (PMC10745504; doi:10.3390/microorganisms11122914)

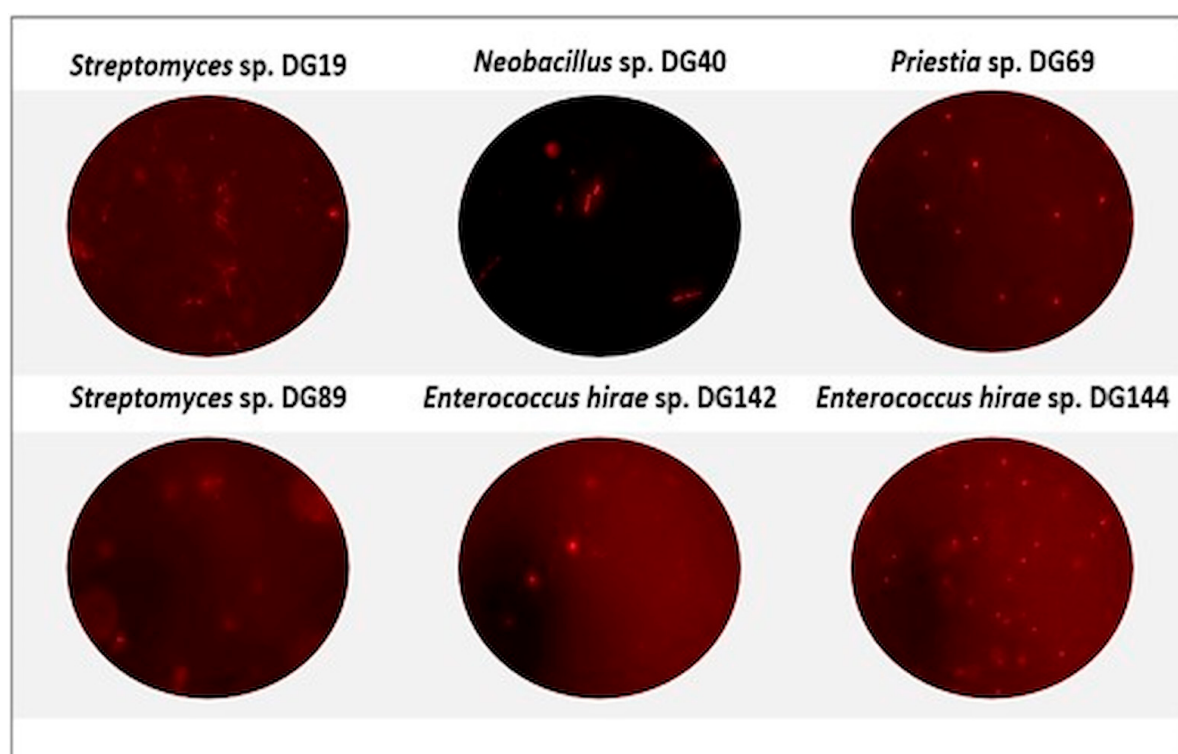

Figure S1: Visualization of PHA granules in isolates via confocal microscopy.

Supplement: Supplementary file 1 [file microorganisms-11-02914-s001.zip › microorganisms-2713747-supplementary.pdf]
